# A novel neutralizing monoclonal antibody targeting the N-terminal domain of the MERS-CoV spike protein

**DOI:** 10.1038/emi.2017.18

**Published:** 2017-05-24

**Authors:** Yingzhu Chen, Shuai Lu, Hao Jia, Yao Deng, Jianfang Zhou, Baoying Huang, Yueyang Yu, Jiaming Lan, Wenling Wang, Yongliang Lou, Kun Qin, Wenjie Tan

**Affiliations:** 1 School of Laboratory Medicine and Life Science, Institute of Medical Virology, Wenzhou Medical University, Wenzhou 325035, China; 2 Key Laboratory of Medical Virology, National Health and Family Planning Commission, National Institute for Viral Disease Control and Prevention, China CDC, Beijing 102206, China

**Keywords:** coronavirus, mAb, MERS-CoV, neutralizing, NTD, RBD

## Abstract

Middle East respiratory syndrome coronavirus (MERS-CoV) has caused fatal infections, some through hospital-acquired transmission, in affected regions since its emergence in 2012. Although the virus is not pandemic among humans, it poses a great threat to public health due to its zoonotic origin. Thus, both preventative and therapeutic countermeasures are urgently needed. In this study, we discovered a panel of neutralizing monoclonal antibodies (mAbs) against MERS-CoV, which mapped to a wide range of regions on the spike (S) protein of the virus. In addition to mAbs with neutralizing epitopes located on the receptor-binding domain, one mAb, 5F9, which binds to the N-terminal domain (NTD) of the MERS-CoV S1 subunit, showed efficient neutralizing activity against the wild-type MERS-CoV strain EMC/2012, with a half maximal inhibitory concentration of 0.2 μg/mL. We concluded that a novel neutralizing epitope for MERS-CoV also resides on the NTD of the S protein, indicating that the NTD might be important during the viral infection process. Our findings have significant implications for further vaccine design and for the development of prophylactic and therapeutic monoclonal immunotherapies against MERS-CoV infection.

## INTRODUCTION

Middle East respiratory syndrome coronavirus (MERS-CoV), a novel lethal human pathogen, has led to 1879 laboratory-confirmed cases of infection with an approximate fatality rate of 36% since its identification in Saudi Arabia in 2012.^1^ The symptoms caused by MERS-CoV are similar to the symptoms of severe acute respiratory syndrome coronavirus (SARS-CoV), manifesting as an acute and severe lower respiratory illness with extrapulmonary involvement, including vomiting, diarrhea and renal failure.^2, 3^ Infections have been confirmed in 27 countries, with the most cases occurring in the Middle East, followed by the recent outbreak in the Republic of Korea.^4, 5^ Serological and virological surveys have indicated that dromedary camels are likely the major reservoir of MERS-CoV.^6, 7, 8^ Although some human-to-human transmission cases, including contact with health care workers and family members, have been reported,^9, 10, 11, 12^ it remains possible that the virus could acquire an adaptive mutation during repeated interspecies transmission events. Due to its potential threat, MERS-CoV has been listed as a Category C Priority Pathogen by the National Institute of Allergy and Infectious Diseases. However, neither licensed vaccines nor antiviral drugs against MERS-CoV have been approved for clinical use. Efficient countermeasures against this virus are urgently needed.

**Figure 1 fig1:**
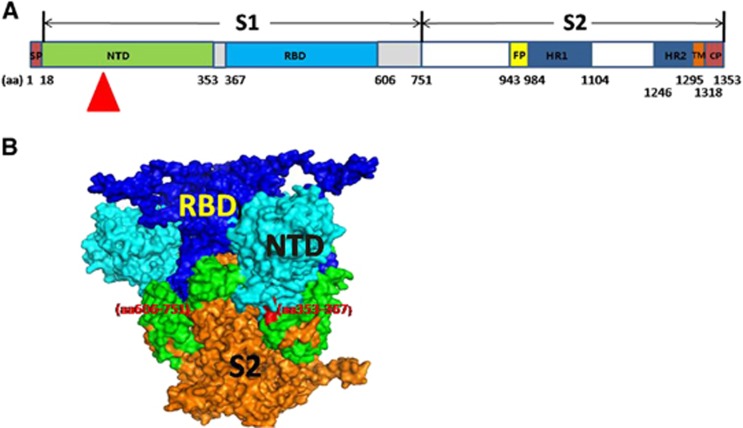
Schematic diagram and analogous three-dimensional (3D) structure of the MERS-CoV S protein. (**A**) Amino acid sequences of the recombinant proteins (rNTD, rRBD, rS1, rS2 and rS) evaluated in this study. The NTD region of focus in this study is indicated by a red triangle. (**B**) The 3D structure of the MERS-CoV S protein was predicted using PyMOL, and the side view or transverse view is shown based on the trimeric S structure of HKU1.^18^ N-terminal domain (NTD), RBD, S2 and the 367–606 and 606–751 aa regions are colored in light blue, dark blue, orange, red and green, respectively.

MERS-CoV is an enveloped positive-sense single-stranded RNA virus that belongs to the lineage C β-CoV genus. The MERS-CoV genome is ~30 kb and encodes the 5′-replicase structural protein (spike-envelope-membrane-nucleocapsid)-poly (A)-3′.^13, 14, 15^ The highly glycosylated spike (S) protein mediates viral infections and is a primary determinant of cell tropism and pathogenesis. It assembles as a trimer on the viral particle surface and contains two functional subunits. The S1 subunit (residues 1–751) is mainly responsible for mediating viral particle attachment to the cell surface and is dependent on the dipeptidyl peptidase 4 (DPP4) receptor.^16, 17^ The S2 subunit (residues 752–1353) facilitates the subsequent fusion of the virus with the host cell membrane (Figures 1A and 1B). To deliver the viral nucleic acid into the host cell, the S1 subunit binds to the cellular receptor and triggers conformational changes in the S2 subunit, which then inserts its fusion peptide into the target cell membrane to form a six-helix bundle fusion core that prepares the viral and cell membranes for fusion.^19^

The receptor binding domain (RBD) of the S1 subunit serves as a determinant region for the production of MERS-CoV neutralizing antibodies^20, 21, 22, 23, 24, 25, 26, 27^ and has been the target for the development of a number of promising MERS-CoV vaccine candidates.^27, 28, 29, 30, 31, 32^ The MERS-CoV RBD maps to a 200–300 residue region spanning residues 358–588, 367–588, 367–606, 377–588 and 377–662, which are located in the S1 subunit C-terminal domain.^16, 17, 33, 34, 35, 36, 37, 38, 39, 40^ The specific interaction between MERS-CoV RBD and its receptor DPP4 (also known as CD26) has also been revealed at the atomic level by structural analysis.^16, 17^ Accumulating evidence shows that all the neutralizing antibodies with therapeutic potential against MERS-CoV interfere with the association of RBD with DPP4.^20, 21, 22, 23, 24, 25, 26, 27^ In addition to the immunogenic RBD, the undefined N-terminal domain (NTD) accounting for a large portion of S1 may serve as a functional component of the MERS-CoV S1 subunit. NTD has been recognized as the receptor-binding site in several CoVs, including murine hepatitis virus.^41^ In this study, we discovered a neutralizing mAb that specifically recognizes the MERS-CoV S protein NTD. This study highlights the importance of the region in the viral infection process, which may enable us to better understand the underlying neutralizing mechanism of natural MERS-CoV infection. These findings pave the way for MERS-CoV vaccine and immunotherapy development.

## MATERIALS AND METHODS

### Ethics statement

Female BALB/c mice aged six to eight weeks were used for mAb production. The animal studies were performed in strict compliance with the Guide for the Care and Use of Laboratory Animals of the People’s Republic of China. The study protocol was approved by the Committee on the Ethics of Animal Experiments of the Chinese Center for Disease Control and Prevention.

### Cell lines, virus and reagents

Vero E6, 293FT and Huh7.5 cell lines were cultured in Dulbecco’s Modified Eagle medium (DMEM) supplemented with 10% fetal bovine serum (FBS), 1% penicillin-streptomycin (P/S), 1% l-glutamine and 1% HEPES. Non-essential amino acids (1%) were added to Huh7.5 cell cultures. SP2/0 cells were grown in RPMI 1640 containing 20% FBS, 1% P/S, 1% l-glutamine and 1% HEPES. After fusion with immunized mouse spleen cells, the hybridomas were cultured in DMEM with 20% FBS, 2% hypoxanthine aminopterin thymidine (HAT) and 1% P/S. All cells were cultured at 37 °C with 5% CO_2_. All reagents were purchased from Gibco (Life Technologies, Grand Island, NY, USA), except for DMEM, which was obtained from HyClone (Life Technologies, South Logan, UT, USA).

The MERS-CoV (HCoV-EMC/2012) strain was kindly provided by Professor Ron Fouchier (Erasmus Medical Centre, Rotterdam, Netherlands). The plasmids PNL4-3.luc.R^-^E^-^ (a plasmid encoding an envelope-defective and luciferase-expressing HIV-1 genome) and pVRC-MERS-S (a plasmid encoding the spike protein of MERS-CoV) used for the MERS-CoV pseudovirus package were developed in our laboratory.

The baculovirus-insect cell sf9-derived recombinant MERS-CoV (HCoV-EMC/2012) S protein including rS (amino acids (aa) 1–1297) (40069–V08B), rS2 (aa 726–1296) (40070–V08B) and rS1 (40069–V08H) (aa 1–725) was purchased from SinoBiological Inc. (Beijing, China). The recombinant RBD (rRBD) (aa 367–606) and NTD (rNTD) (aa 18–353) expressed in a baculovirus system were kindly provided by Professors Jinghua Yan and George F Gao (Institute of Microbiology, Chinese Academy of Science, Beijing, China). The mouse mAb Isotyping Kit was purchased from Pierce (Life Technologies). The HiTrap protein G columns, Protein A sensor chip, 10 × PBS-P buffer (pH 7.4) and 10 mM glycine-HCl (pH 1.5) were purchased from GE Healthcare (Pittsburgh, PA, USA).

### Mouse immunization and mAb generation

Mice were immunized with 35 μg MERS-CoV rS combined with 150 μL Freund’s complete adjuvant (Sigma, St Louis, CA, USA) via subcutaneous immunization and boosted twice at 2-week intervals beginning three weeks after the initial immunization. The mice were killed 3 days after the last immunization, and their splenocytes were fused with mouse myeloma cells.

The mAbs were generated as previously described.^42^ Cells collected from the spleens of killed animals were fused with cultured SP2/0 cells at a 10:1 ratio in the presence of PEG1450 (Sigma). HAT selection medium was used for the fused hybridoma cultures. After a 2-week incubation, the positive hybridomas were selected using rS-coated ELISA, and the positive clones were subjected to limited dilutions and downstream validation. For large-scale mAb production, ascites fluid from mice inoculated with the hybridomas was collected and purified using a caprylic acid-ammonium sulfate precipitation assay and HiTrap Protein G columns with the AKTA system (GE Healthcare). Isotype classification of the purified mAbs was performed using the Pierce Rapid ELISA Mouse mAb Isotyping Kit (Pierce, Rockford, IL, USA) according to the manufacturer’s instructions.

### ELISA assay

To precisely determine the binding regions of the mAbs targeting the S protein of MERS-CoV, truncated S fragments (rS1, rS2, rRBD and rNTD) and a panel of 43 peptides (Supplementary Table S1) spanning the entire MERS-CoV NTD were used as coating antigens in an indirect ELISA. Briefly, 96-well ELISA plates (Corning, Shanghai, China, Asia) were coated with the recombinant proteins (1 μg/mL) or 18 aa peptides (2.5 μg/mL) overnight at 4 °C. After blocking, the mAbs were added to the wells and incubated for 1.5 h at 37 °C. The plates were then incubated with HRP-conjugated secondary antibody for 1 h at 37 °C, and the binding activity was determined at 450 nm using a plate reader (Multiskan MK3). An anti-influenza virus N9-specific mAb was used in all assays as an unrelated negative control, and pre-fusion mouse serum was used as a positive control.

### Affinity measurement by Biacore

The binding kinetics and affinity of the mAb 5F9 to purified rNTD were analyzed by surface plasmon resonance (SPR) (Biacore T200, GE Healthcare). The mAb 5F9 was immobilized to a Protein A sensor chip via binding to the Fc region in 1 × PBS-P+buffer (pH 7.4). Different concentrations of purified rNTD (60, 30, 15, 7.5 and 3.25 nM) were run at a flow rate of 10 μL/min in PBS-P+buffer. The surface was regenerated with 10 mM glycine-HCl (pH 1.5). Sensorgrams were fit with a 1:1 binding model using BIA Evaluation software (GE Healthcare).

### Western blot analysis

Purified MERS-CoV rS and rNTD were analyzed on 12% SDS–polyacrylamide gels supplemented with 2 × SDS after boiling for 15 min. The denatured proteins were then transferred to a nitrocellulose membrane, blocked with 5% non-fat milk in PBST for 1 h at room temperature, and probed overnight with 1 μg/mL mAb and pre-fusion mouse serum (1:10 000 dilution) at 4°C. The membranes were incubated with DyLight 800 IgG secondary goat anti-mouse IgG (LI-COR Biosciences, NE, USA) and scanned using the Odyssey Infrared Imaging System (LI-COR Biosciences, Lincoln, NE, USA).

### Neutralization test

The neutralizing activity of the selected mAbs was initially determined using pseudoviruses as described previously.^28^ Briefly, 293FT cells were co-transfected with the plasmids PNL4-3.luc.R^-^E^-^ and pVRC-MERS-S, and culture supernatant containing sufficient pseudotyped MERS-CoV was collected 48–72 h post-transfection. Subsequently, DPP4-expressing Huh7.5 cells, upon reaching a density of 5 × 10^3^/well in a 96-well plate, were infected with 200 TCID_50_ (50% tissue culture infective dose) pseudovirus MERS-CoV in the presence or absence of mAbs. The culture medium was renewed with fresh medium containing 2% FBS 18–20 h post-infection, and luciferase activity was determined after an additional 48 h incubation using an Infinite M1000 illuminometer.

The neutralizing activity of the mAbs against live MERS-CoV was also determined in DPP4-expressing Vero E6 cells. Upon reaching a density of 5 × 10^4^/well in a 12-well plate, cell monolayers were infected with 30–35 plaque-forming units (PFU) of live virus in the presence or absence of the mAb. After three days of incubation at 37°C, the inhibitory capacity of the mAbs was assessed by determining the numbers of PFU compared with the potent MERS-CoV anti-RBD and anti-N9 mAbs. All experiments associated with live MERS-CoV were conducted in a BSL-3 laboratory at the National Institute of Viral Diseases Control and Prevention, China CDC.

## RESULTS

### Characterization of the mAbs

**Figure 2:**
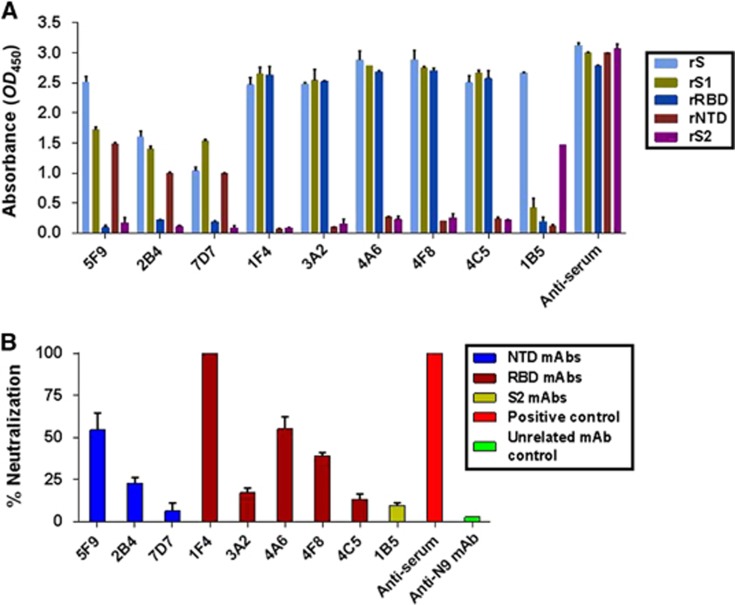
The isolated mAbs exhibited different binding affinities and neutralizing potencies to MERS-CoV. (**A**) These mAbs (5F9, 2B4, 7D7, 1F4, 3A2, 4A6, 4F8, 4C5 and 1B5) were mapped to different regions of the MERS-CoV S protein using ELISA. rS, rS1, rS2, rRBD and rNTD were used at a concentration of 1 μg/mL. (**B**) Inhibition of pseudovirus MERS-CoV entry into Huh7.5 cells by the isolated mAbs. Each of the selected mAbs (1 μg/mL), anti-N9 mAb (1 μg/mL) and anti-serum (1:10 000) were evaluated for their neutralizing activity against pseudotyped MERS-CoV after incubation with 293FT cell surface-expressing MERS-CoV S proteins. Neutralizing activity was defined as occurring when the inhibition percentage exceeded 50%. The results shown are representative of three independent experiments. N-terminal domain, NTD.

To identify novel neutralizing epitopes on the MERS-CoV S protein, a panel of mAbs targeting the S protein (aa 1–1297) was generated using a traditional hybridoma fusion protocol.^42^ Nine rS-ELISA binding mAbs covering the RBD, the NTD and outside these two regions were selected from 37 clones. As shown in Figure 2A and Table 1, all mAbs bound strongly to sf9-derived rS (aa 1–1297) at a concentration of 1 μg/mL. Five of these mAbs (1F4, 3A2, 4A6, 4F8 and 4C5) bound strongly to rRBD (aa 367–606), while one (1B5) and three mAbs (5F9, 2B4 and 7D7) bound to rS2 (aa 753–1353) and rNTD (aa 18–353), respectively.

Next, we assessed the neutralizing activities of the nine selected mAbs against 200 TCID_50_ MERS-CoV pseudoviruses. As shown in Figure 2B, only mAbs 5F9, 1F4, 4A6 and 4F8 exhibited greater than 50% neutralization potency at 1 μg/mL compared to pre-fusion mouse serum (1:10 000 dilution). The rRBD-binding mAb 1F4 showed the strongest neutralizing activity (~100%); another two mAbs (4A6 and 4F8) and one novel rNTD-binding mAb (5F9) exhibited efficient neutralizing activity (over 50%). The other five mAbs, including 1B5 (rS2-binding mAb), exhibited less than 20% neutralization activity. The mAb 5F9 was therefore selected for further characterization and evaluation.

### Binding efficiency of 5F9 to rNTD of MERS-CoV

**Figure 3:**
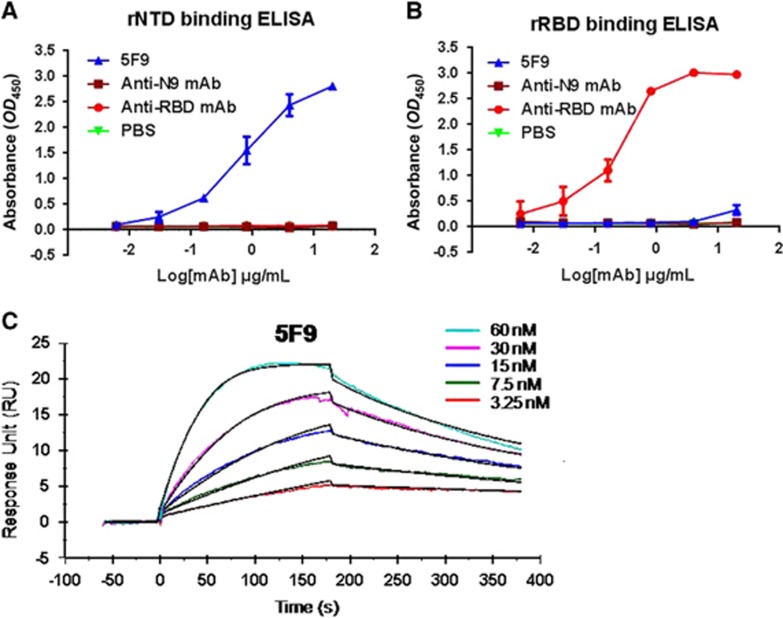
The 5F9 antibody bound to the rNTD with high affinity. Recombinant proteins at 1 μg/mL were used to coat plates overnight at 4 °C, and each of the mAbs was serially diluted in PBS and assessed for binding affinity and specificity to MERS-CoV rNTD (**A**) and rRBD (**B**). (**C**) The binding kinetics between 5F9 and rNTD using SPR. The Ka and KD values were calculated from sensograms using five rNTD concentrations (60, 30, 15, 7.5 and 3.25 nM). The results shown are representative of three independent experiments. Anti-RBD, anti-N9 mAb and PBS were used as the positive, unrelated and blank controls, respectively. N-terminal domain, NTD.

To further determine the binding efficiency of mAb 5F9, samples were diluted five-fold and detected using an MERS-CoV rNTD-coated ELISA. The results showed that 5F9 had a high binding affinity for rNTD, with an EC_50_ of approximately 0.85 μg/mL (Figure 3A). Meanwhile, 5F9 exhibited no cross-reaction with rRBD, even at a concentration of 4 μg/mL (Figure 3B). The anti-N9 mAb was evaluated in parallel as an unrelated negative control.

Next, the binding rate constant of the mAb 5F9 to rNTD was determined by SPR. As shown in Figure 3C, 5F9 showed nanomolar affinity for the rNTD (equilibrium dissociation constant (KD) equivalent to 5.42 nM).

### Neutralizing efficiency of 5F9 to MERS-CoV

**Figure 4:**
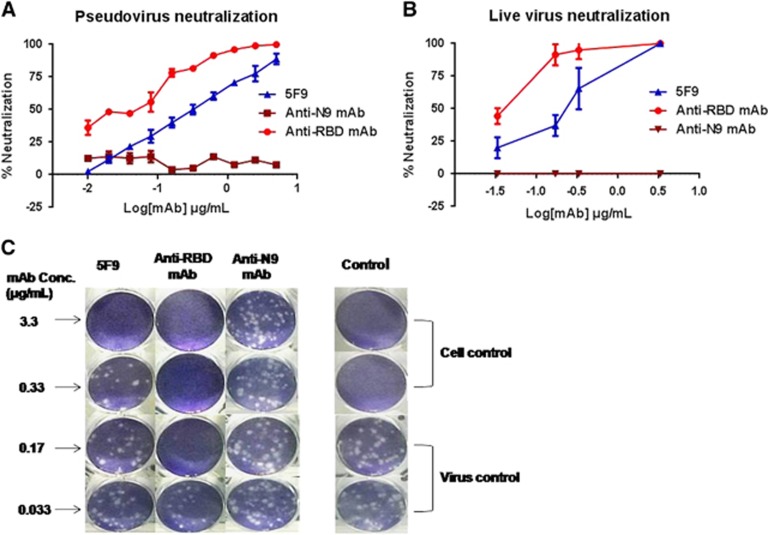
Potent *in vitro* neutralization of MERS-CoV by 5F9 mAb. (**A**) Neutralization of pseudotyped MERS-CoV. DPP4-expressing Huh7.5 cells were cultured with 200 TCID_50_ pseudotyped MERS-CoV in the presence of serially diluted mAbs. The neutralizing percentage was calculated by measuring luciferase expression compared to the pseudovirus-infected cell control. (**B**) Neutralization of live viruses. Different concentrations of the mAbs were pre-cultured with the live viruses (HCoV-EMC) in Vero E6 cell monolayers. The neutralization percentage was evaluated by calculating the decrease in PFU number compared with the virus-infected control. (**C**) PFU images of viral infection in the presence of the mAbs on day 3. The images correspond to the neutralizing percentages in **B**. Approximately 30–35 PFU virus stocks (HCoV-EMC) were used to infect Vero E6 cells in a 12-well plate with or without mAbs. The results shown are representative of three independent experiments. Anti-RBD and anti-N9 mAbs were used as positive and unrelated controls, respectively.

Previous results showed that 5F9 exhibited approximately 60% neutralizing activity toward pseudovirus MERS-CoV at a concentration of 1 μg/mL. We determined the lowest concentration of the mAb that can effectively inhibit pseudovirus MERS-CoV entry using two-fold dilutions of the mAbs. The results showed that the neutralizing activity of 5F9 was dose dependent, and the IC_50_ was approximately 0.24 μg/mL. The IC_50_ of anti-RBD mAb (0.02 μg/mL) was 10-fold lower than the IC_50_ of 5F9 (Figure 4A). Therefore, 5F9 showed efficient neutralizing activity and was further characterized.

The neutralizing potency against live MERS-CoV (strain HCoV-EMC/2012) was also determined. As shown in Figure 4B, mAb 5F9 neutralized the live MERS-CoV infection, with the same IC_50_ of 0.2 μg/mL. Images of the reduced PFU formation, corresponding to the live MERS-CoV neutralizing percentages, are shown in Figure 4C. Together, the neutralization data indicate that MERS-CoV NTD may play an important role in the viral infection process.

### Preliminary epitope analysis of the mAb 5F9

**Figure 5:**
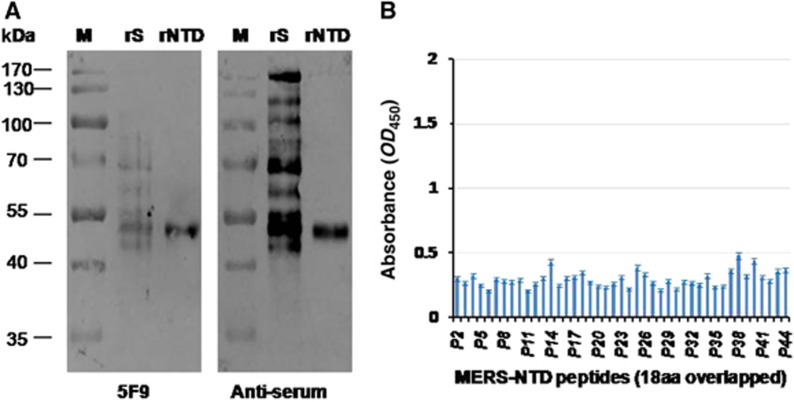
Binding abilities of 5F9 to denatured MERS-CoV rS, rNTD and synthetic peptides spanning the NTD. (**A**) Western blot analysis of mAb 5F9 bound to denatured rS and rNTD. Lane rS, 1 μg denatured rS protein (aa 1–1297); Lane rNTD, 1 μg denatured rNTD (aa 18–353). Pre-fusion mouse serum (anti-serum) was used as a positive control. (**B**) Binding of 5F9 to overlapping 18 aa peptides. P2 to P44 (18 aa peptides) correspond to numbers 2 to 44 in Supplementary Table S1. Each peptide was coated onto a 96-well plate at 250 ng/well, and the mAb was evaluated at a concentration of 1 μg/mL. The results shown are representative of three independent experiments. N-terminal domain, NTD.

To further characterize the binding abilities of mAb 5F9 to the MERS-CoV NTD (aa 18–353), we employed a Western blot assay in which the rNTD was denatured to determine if the epitope was conformational or linear. The results showed that mAb 5F9 efficiently bound both the MERS-CoV rNTD (aa 18–353) and rS (Figure 5A). An 18 aa peptide ELISA was performed to locate the binding site of 5F9. The results showed that 5F9 bound very weakly to these peptides (*OD*_450_<0.5) (Figure 5B, Supplementary Table S1). These results suggest a possible linear binding epitope between 5F9 and the NTD, although the specific region was not clearly determined.

## DISCUSSION

Several studies have demonstrated that most of the potent neutralizing mAbs against MERS-CoV derived from both human and murine origins target the RBD region of the S protein.^20, 21, 22, 23, 24, 25, 26, 27^ In addition, these mAbs have shown promising therapeutic value in animal models.^23, 24, 26, 27^ RBD-based vaccines have also elicited strong humoral immune responses using different immunization strategies.^27, 28, 29, 30, 31, 32^ Our findings demonstrate that the mAb 5F9 antibody specifically targets the MERS-CoV NTD and exhibits efficient neutralizing activity against MERS-CoV, although its *in vitro* neutralizing potency (IC_50_ 0.2 μg/mL) was approximately 10-fold lower than the potency of the RBD-targeting mAbs. Thus, this mAb warrants further development and evaluation in animal models to provide alternatives for MERS-CoV immunotherapy should the virus mutate and no longer remain susceptible to RBD-specific mAb treatment.

The surface S segment is the major CoV structural protein, and maintaining the quaternary nature of the S1–S2 complex is vital for viral infections. Recently, one study showed that SARS-CoV infection is affected by the inactive-to-active state transition of the S glycoprotein trimer. In addition, the loops connecting the NTD to the C-terminal domain (CTD) 1 and CTD1 to CTD2 were found to play key roles in the conformational switch.^43^ Lassa virus (LASV) envelope glycoprotein complex (GPC) is another class I viral fusion protein that initiates infection by processing into GP1, GP2 and an unusual stable signal peptide (SSP). GP1 is responsible for receptor binding, while GP2 mediates the fusion of the virion with a cell membrane. Previous studies demonstrated that gaps in the glycan shield of LASV GPC permitted neutralizing antibodies to interact simultaneously with the N-terminal extension of GP1 and either the fusion loop or the T-loop of GP2, inhibiting the structural alterations required for fusion.^44^ These discoveries showed that maintaining glycoprotein structural integrity during viral infection is necessary and that neutralizing mAbs targeting different surface glycoprotein epitopes can interfere with structural state switching. As shown in Figures 1A and 1B, 1structural docking of the MERS-CoV S protein revealed the NTD and C-terminal RBD of the S1 subunit, which form extensive quaternary interactions with each other. These features may imply that certain epitopes on the NTD of MERS-CoV S1 are structurally or functionally close to the ones on the RBD and that the mAbs targeting these epitopes can interfere with receptor binding or the post-fusion stage.

The glycan composition of class I envelope glycoproteins can shield a virus against the neutralizing effect of a mAb. A neutralizing mAb against the HIV-1 envelope glycoprotein was found to bind to the residues and glycans on gp41 and gp120, thereby precluding the CD4- and/or co-receptor-induced conformational changes required for membrane fusion.^45^ The S proteins of the *Coronaviridae* family, consisting of four genera (α-, β-, γ- and δ-CoVs), are also class I envelope glycoproteins, and all use the S1 protein RBD or NTD to interact with cellular receptors or co-receptors for viral attachment.^46^ To the best of our knowledge, most α-CoVs and some β-CoVs, including SARS-CoV, MERS-CoV and HCoV-HKU1, use protein moieties as their recognition receptors, and their main functional RBDs are located in the CTD of S1. In contrast, the RBDs of bovine CoV, human CoV OC43 and one avian γ-CoV are located in the NTD, and sugar moieties are the main receptors. More notably, the NTD and CTD of two α-CoVs, porcine epidemic diarrhea CoV and transmissible gastroenteritis, might both play vital roles in viral infection.^47, 48^ The NTD of MERS-CoV is also glycosylated, and the neutralizing mAb 5F9 may bind to the glycan or other residues of the NTD, stabilizing the pre-fusion conformation of the S protein and the S1–S2 interaction, thereby precluding the DPP4- and/or co-receptor-induced conformational changes required for membrane fusion.

Continuing human infection with MERS-CoV is a global public health concern that highlights the need for efficient countermeasures. Currently, various vaccines and therapeutic antibodies against MERS-CoV are under development, and most have targeted the MERS-CoV RBD because it contains the most variable regions. Multiple evaluations of the full length S-based or truncated S1-based vaccines indicate that an increase in epitope range would be important for future MERS-CoV vaccine designs.^49, 50^ Our study suggested that the NTD, which contains the neutralizing epitope, should be considered an immunogenic component. Thus, we propose that combining the most promising vaccine candidate with the NTD would not only improve the protective effects but also reduce the likelihood of possible resistance development via mutation.

## Figures and Tables

**Table 1:** Characterization of the mAbs against MERS-CoV in this study

**mAb**	**Isotype**	**Binding region**					**Neutralization of MERS-CoV pp**^a^
		**RBD**	**NTD**	**S1**	**S2**	**S**	
5F9	IgG2b, κ	−	+	+	−	+	50%–90%
2B4	IgG2a, κ	−	+	+	−	+	<50%
7D7	IgG2b, κ	−	+	+	−	+	<50%
1F4	IgG2a, κ	+	−	+	−	+	>90%
3A2	IgG2b, κ	+	−	+	−	+	<50%
4A6	IgG2b, κ	+	−	+	−	+	∼50%
4F8	IgG2b, κ	+	−	+	−	+	<50%
4C5	IgG2b, κ	+	−	+	−	+	<50%
1B5	IgG2a, κ	−	−	−	+	+	<50%

Abbreviation: N-terminal domain, NTD.

a The pseudovirus MERS-CoV was neutralized by mAb at a concentration of 1 μg/mL.
